# IT HURTS AS IF: PAIN LANGUAGE, VISUAL CHARACTERIZATION, AND STORY-TELLING IN HMONG OLDER ADULTS

**DOI:** 10.1093/geroni/igz038.275

**Published:** 2019-11-08

**Authors:** Maichou Lor, Xia Vang, David Rabago, Roger Brown, Miroslav Backonja

**Affiliations:** 1 Columbia University, New York, New York, United States; 2 University of Wisconsin-Madison, School of Medicine and Public Health, Department Family Medicine and Community Health, Madison, Wisconsin, United States; 3 University of Wisconsin-Madison, School of Nursing, Madison, Wisconsin, United States; 4 School of Medicine and Public Health, Department of Neurology, Madison, Wisconsin, United States

## Abstract

Culture and language affect pain reporting, diagnosis, and treatment. Ethnic subgroup populations, such as the Hmong, are understudied in pain research. The study’s purpose is to qualitatively understand older Hmong adults’ pain expression and their pain communication with providers. Sixty-seven participants were recruited from one healthcare system and community. A directed content analysis revealed that all Hmong participants describe pain using stories with reference to the temporal context, causal attribution, co-occurring symptoms or related experiences, magnitude, and consequences of pain. Several participants also characterized their pain by associating it with visual metaphors as objects and animals. Some participants shared that their stories are often underappreciated by providers, and are therefore not understood by providers. This leads to subsequent feelings of stress, not receiving needed medication or other healthcare, and having less frequent contact with providers or switching providers. These findings have implications for more culturally attentive and appropriate pain care.

